# Construction of a ‘Simple, Fast and Accurate’ Evaluation Method for Profile Control and Plugging Effect of Gel Plugging Agent Based on Simulations

**DOI:** 10.3390/gels11020115

**Published:** 2025-02-06

**Authors:** Zengbao Wang, Junjie Jiang, Weian Huang, Yuwei Gan, Yingrui Bai

**Affiliations:** 1 School of Petroleum Engineering, China University of Petroleum (East China), Qingdao 266580, China; jjunjie2000@163.com (J.J.); masterhuang1997@163.com (W.H.); hh498082@163.com (Y.G.); smart-byron@163.com (Y.B.); 2 Key Laboratory of Unconventional Oil & Gas Development, China University of Petroleum (East China), Ministry of Education, Qingdao 266580, China

**Keywords:** gel plugging agent, evaluation method, experimental design, cohesive strength, adhesive strength, degree of damage

## Abstract

At present, the evaluation perspective of the gel plugging agent assessment method is incomprehensive, due to which the experimental results deviate from the field data. By analyzing the current indoor evaluation methods and the factors controlling the sealing capability of gel plugging agents, an experimental device and method for evaluating the blocking effect of oilfield gel plugging agents has been designed. In contrast to traditional assessment methods, the proposed approach offers advantages such as simple operation, rapid experimentation, and accurate results. The experimental results show that gels selected using conventional methods are inconsistent with the results of plugging displacement tests. This discrepancy can be attributed to the fact that these methods focus solely on cohesive strength while neglecting adhesive strength. Considering that the evaluation perspective of conventional methods is relatively limited, an evaluation method for the sealing effect of the plugging agent was developed. This method comprehensively incorporates factors such as cohesion strength, adhesion capability, shear resistance, and the long-term anti-dehydration performance of the gel. The evaluation results of the method were consistent with the results of the plugging displacement experiments. The newly constructed method defines *Γ* as the comprehensive evaluation parameter for the gel. A new experimental system with a comprehensive evaluation index (*Γ*) of 8.97 Pa^2^ was selected. After the profile control of the system, the effluent ratio of the high and low permeability layers reached 1:9, and its erosion resistance was greater than 20 PV. Meanwhile, the profile control effect was also stable. Through verification based on field data, the injection pressure of the system optimized by the proposed method was found to be 2.5 times higher than that of the original system. Meanwhile, the plugging validity period was >2 times of the original system. The test results were consistent with the plugging capability evaluation index. In summary, the performance evaluation method of the designed gel plugging agent was reasonable in principle and the results were accurate and reliable. Therefore, it is considered to be of guiding significance for the selection of efficient profile control plugging agents in oilfields.

## 1. Introduction

**Figure 1:**
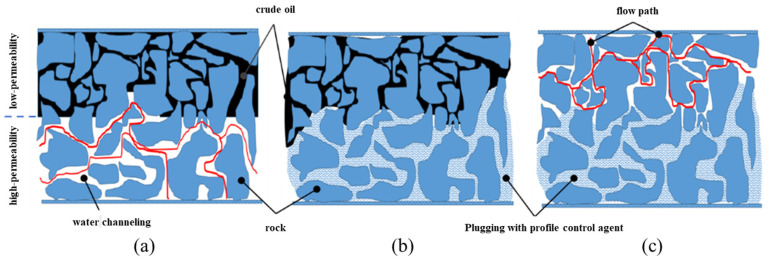
Schematic of the regulation of plugging agent to improve water flooding flow field. (**a**) Occurrence of water channeling; (**b**) Utilization of profile control agent; (**c**) Turning of water flow.

With the continuous water injection development of oilfields, the heterogeneity of the formation has aggravated [1]. Meanwhile, some production wells have reached a high water-cut stage, and the development benefits are getting worse and worse [2]. The early reaching of high water cuts in production wells is mainly caused by water channeling [3]. Generally, the fluid in the reservoir will flow out preferentially along the channel with the minimum flow resistance so that the injected water will form a water channeling passage in the high-permeability layer [4], which will form the phenomenon of “repeated flushing of the high-permeability channel and non-entry of the low-permeability channel”. In addition to altering the water injection pattern to address water channeling, profile modification and water plugging are also widely adopted methods [5]. The profile-controlling and water-plugging agent is a special material used to adjust the water absorption profile and realize the diversion of liquid flow to deal with water channeling [4,6,7] (see Figure 1). Among many profile-controlling and water-plugging agents, gel plugging agents are widely used in oilfields due to their adjustable strength, good injection performance, and simple construction [8]. For example, Li et al. [9] developed a high-elastic expansion particle as well as the phenolic gel system, which was used in nine wells of the Jilin oilfield, with a cumulative reduction in water loss of 56,900 m^3^ and a cumulative oil increase of 3200 tons. The overall profile-controlling and plugging effect was obvious. Li et al. [10] research on polymer gel plugging agents, primarily based on acrylic resins (MMA). The gel system exhibits a plugging strength of 6 × 10⁴ mPa·s and a plugging rate of 99.79%, demonstrating exceptional performance in water control and profile modification.

In field applications, the profile-controlling effect of gel is affected by many factors. For example, the gel is sheared by the ground equipment and the migration of formation [11], resulting in the degradation of some polymers. Under strong water flooding, some of the gel is discharged without bonding with the reservoir [12]. These factors will reduce the plugging effect of the gel and affect the effectiveness of the on-site process implementation. Therefore, it is required that the gel system should have good strength, shear resistance, and adhesion capacity. However, how to properly characterize, evaluate, and screen the gel system is still not studied in detail in the literature.

At present, the most commonly used performance evaluation methods of gel plugging agent include breakthrough vacuum degree method [13], transition pressure method [14], cylinder immersion depth method [15], void resistance factor method [16], visual code method [17], rheological parameter method [18], rotary viscometer method [19], falling ball viscosity method [20], capillary viscometer method [21], flow length evaluation method [22], and gel strength rank (GSR) index method [23]. In the breakthrough vacuum degree method, the maximum degree of vacuum (BV) of air breaking through the gel using negative pressure is measured to characterize the strength. The transition pressure method compares the gel strength by recording the pressure at which the gel overcomes the intermolecular binding from the flow state. The cylinder immersion depth method involves recording the depth of the cylinder sinking into the gel within the specified time, which is represented as the gel strength index. The strength index is inversely proportional to the gel strength. By comparing the same volume of gel and polymer solution through the same length and permeability of the sand filling tube time, the method of void resistance factor method is used to reflect the relative strength of colloid and polymer solution. In the visual code method, the sample tube is inverted to observe the shape of the gel in the tube to intuitively evaluate the gel strength. This method is simple, convenient, and applicable to a wide range of conditions, and is the most commonly used method. In the rheological parameter method, the storage modulus of the gel system was measured using a rheometer to compare the strength of different gel formulations. The rotary viscometer method is used for characterizing the strength of gel by measuring the ease of flow of gel at a certain temperature and shear rate using a rotary viscometer. Similarly to the cylinder immersion depth method, the falling ball viscosity method is suitable for high strength gels. The capillary viscometer method is used for indirectly measuring the strength of gel by measuring the time required for inhaling a unit volume of gel in a capillary. By comparing the length of the gel after vertical and horizontal placements, the flow length evaluation method defines a criterion for evaluating the colloidal strength. The GSR index method involves measuring the strength of a gel by determining the time it takes to pass through a length of capillary viscometer.

Among them, the first four methods mainly characterize the capability of the gel plugging agent to resist shear breaking, while the latter seven methods mainly show the adhesive capability of the gel plugging agent. However, the effectiveness of a gel plugging agent needs to have both good strength and adhesion capability [24]. The single evaluation method is not used in the actual application in oilfields and lacks scientific basis and practicability [25,26]. Although the plugging capability of a gel plugging agent, evaluated by a core tube displacement test, can be closely combined with the actual construction site, the operation is complicated and the experimental time is long, which are not suitable for the optimization of the formulation and the screening of the batch gel plugging agent [27].

With this backdrop, considering the strength, adhesion, and anti-shearing capability of the plugging gel, an experimental device and a characterization method for evaluating the plugging effect of the gel plugging agent are designed, which are simple to operate, fast in experiment and accurate in evaluation. A more realistic comprehensive evaluation index (defined herein as $\Gamma =\frac{\delta \times \eta }{\lambda }$) is established to characterize the plugging effect of the gel plugging agent on the reservoir. Finally, the rationality, accuracy, and reliability of the experimental method are established through heterogeneous reservoir plugging displacement experiments and field tests. It could provide some effective theoretical basis for field evaluation and optimization of plugging agents.

## 2. Results and Discussion

### 2.1. Screening Using Conventional Test Methods

In the study of indoor synthetic gel, the visual code method [14] and the breakthrough vacuum method are generally used to quickly evaluate the gelling performance of the gel [28,29,30,31]. The former can directly determine the gelling time and gelling strength semi-quantitatively by observing the gelling state of the water shutoff agent after inversion, whereas the latter only needs to use simple instruments such as a vacuum pump and suction filter bottle to reflect the strength of the gel. During the breakthrough vacuum method, when the degree of vacuum increases to the maximum and air breaks through the gel, the BV reading is measured to do further calculations. Both methods have the characteristics of simple operation and are fast and convenient. Based on the visual code method, three gel systems with the same gel strengths (see Table 1) were selected for comparative evaluation, and the breakthrough vacuum of the gel system was determined.

**Table 1:** Comparison of different gel systems for evaluation.

Gel System	Composition of Gel System	The Gel Strength Level Obtained by Visual Code Method	Breakthrough Vacuum Degree, MPa	Remark
Gel system 1	3000 mg/L HPAM + 0.3% phenolic resin + 0.3% organic chromium crosslinking agent + 0.15% crosslinking accelerator	H	−0.094	The system used before the mine
Gel system 2	3000 mg/L HPAM + 0.3% phenolic resin + 0.15% Resorcinol + 0.3% hexamethylenetetramine + 0.15% crosslinking accelerator	H	−0.066	The present system of ore field
Gel system 3	3000 mg/L HPAM + 0.3% amino resin + 0.15% crosslinking accelerator	H *	−0.063	Comparison system

**Figure 2:**
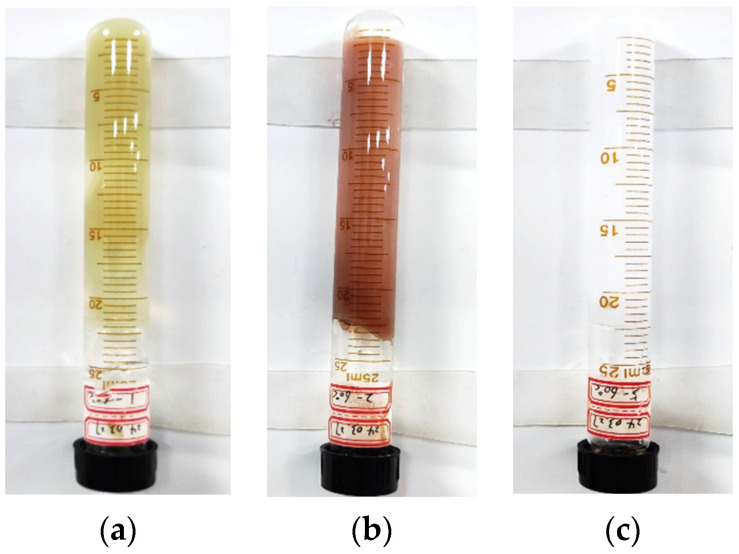
Gelation morphologies of the gels. (**a**) Gel system 1; (**b**) Gel system 2; (**c**) Gel system 3.

* description: visual code method gel strength level H—when the sample bottle is vertically inverted, only the surface of the gel is slightly deformed (Figure 2).

According to the results for the three gel systems (as shown in Table 1), the gel-forming strength of the three gel systems obtained by the visual code method was consistent. However, Gel system 1 exhibited the highest breakthrough vacuum strength, and therefore, the most preferable result was Gel system 1. However, in the field application, it was found that the sealing effect of the preferred Gel system 1 was not ideal. Due to the reason that the adhesion effect between the gel and the reservoir pore throat was not considered, the plugging period was short and it was easy to be destroyed by a subsequent water injection. The reservoir profile control capability was not up to the expectations, and the optimization results of the method were inconsistent with the field application results.

### 2.2. Comparison of Actual Plugging Capability of Gel System

**Figure 3:**
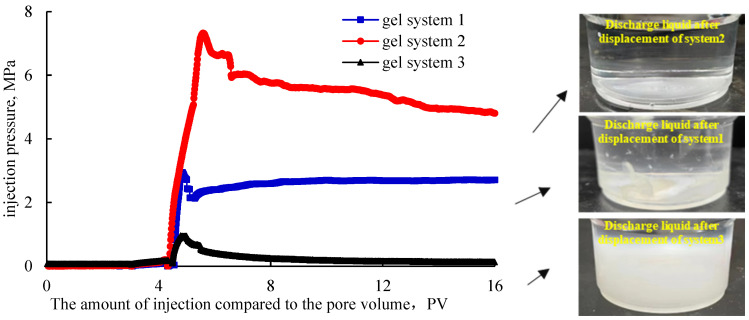
Displacement pressure curve and produced fluid of the gel system after profile control.

Based on the results of the field feedback, the sand-filled pipe or core was used to simulate the application of the field plugging agent and explore the causes of the experimental results. The permeability of the sand-filled pipe filled with 20–80-mesh quartz sand was calculated to be 8.5 μm^2^ using Equation (1). The plugging capabilities of the different gel plugging agents under this permeability were tested. The experimental results of the plugging performance are presented in Table 2, and the displacement pressure curve after plugging is shown in Figure 3.

It can be seen from the results presented in Table 2 that the maximum plugging rate from the three gel systems to the sand-filled pipe and the plugging rate after displacement scouring at 16 PV were all higher than 99%, with little difference in the numerical values. Therefore, the plugging effect from various gel systems could not be effectively distinguished from each other. From the comparison of the results presented in Figure 3, it can be seen that the plugging capacity (maximum breakthrough pressure of 7 MPa) and the erosion resistance (pressure of water injection displacement of 16 PV: 4.8 MPa) of Gel system 2 were obviously better than those of Gel system 1 and Gel system 3. The breakthrough pressure gradient reached the value of 36.57 MPa/m, which was 2.5 times higher than that of Gel system 1 and 7.8 times higher than that of Gel system 3, respectively. The plugging effect was obviously different for the three gel systems. It can be seen from the picture of the discharged liquid after displacement in Figure 3 that, after Gel system 2 was flushed by displacement, almost no gel was washed out from the sand-filling pipe. However, part of Gel system 1 was scoured, sheared, and washed out of the sand-filled tube. Gel system 3 was almost all washed out of the sand-filling pipe, showing the weakest plugging effect. The test results did not agree with the results of the visual code method + breakthrough vacuum method; however, they were basically consistent with the application in the minefield.

**Figure 4:**
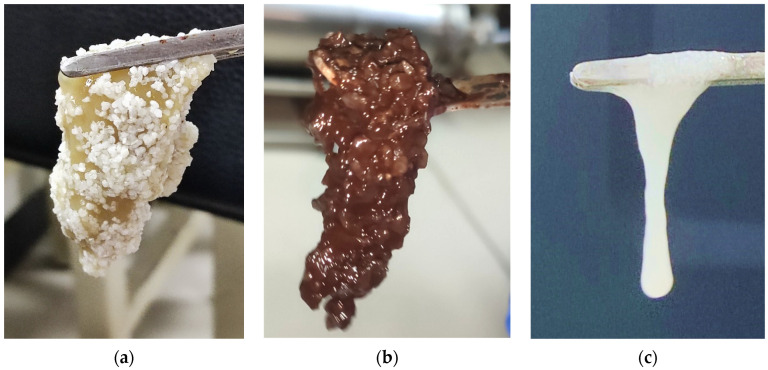
The morphologic diagram of the gel/sand mixtures. (**a**) Gel system 1; (**b**) Gel system 2; (**c**) Gel system 3.

The sand body in the sand-filling pipe was taken out to observe the morphology of the gel/sand mixture. Moreover, the adhesive properties of the gel system were also analyzed. The images of the three types of gel adhesion to sand particles are shown in Figure 4. The sand/gel mixture formed by Gel system 1 was pale yellow. Meanwhile, the residual amount of the colloid is minimal, indicating that most of the gel was displaced early in the displacement experiment due to inadequate adhesive performance. The residual colloid detached from the quartz sand and formed a layered structure due to gravity segregation, further reflecting poor adhesion. The gel’s hanging length is 2.4 cm, with high cohesive strength. On the other hand, the adhesion performance was poor. The sand/gel mixture formed by Gel system 2 was dark brown. The quartz sand and the colloid basically combined into one and were uniformly dispersed. The hanging length was 3.2 cm, which had good adhesion. The sand/gel mixture formed by Gel system 3 was white. The colloid and sand particles were evenly distributed, though the content was small. Only a small amount of mixture could be clamped, and the strength was poor. The results showed that Gel system 2 had excellent adhesion capability compared with the other systems and was more effective in resisting erosion due to water flooding.

**Figure 5:**
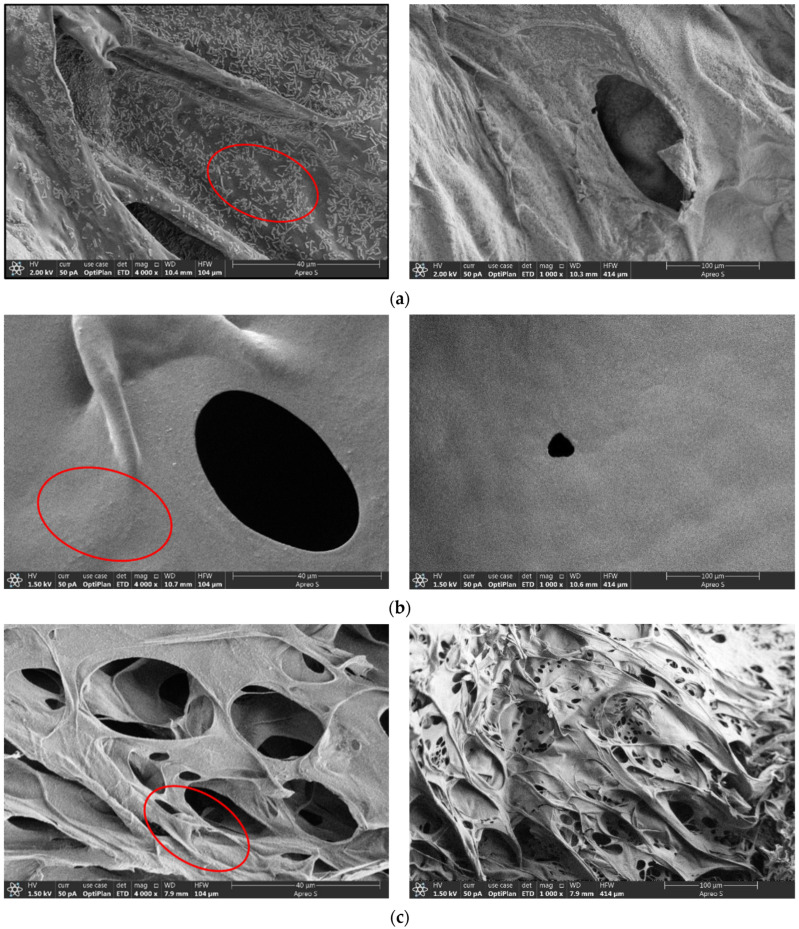
Cryo-SEM images of the three gel systems. (**a**) Cryo-SEM image of Gel system 1; (**b**) Cryo-SEM image of Gel system 2; (**c**) Cryo-SEM image of Gel system 3.

In order to find out the reason why the results about the plugging capabilities of the three gel systems were inconsistent, the three gel systems were placed in a constant-temperature oven for continuous aging for 2 d. The micro-morphology of the gel was analyzed using a freeze scanning electron microscope. It can be seen from the experimental results (Figure 5) that after Gel system 1 formed the gel, the gel became closely connected with itself, forming a cemented structure with high solid strength. However, the surface of the gel had point-shaped or rod-shaped protrusions (Within the ellipse in Figure 5a), so there was a gap when the gel system adhered to the rock surface, which reduced the adhesion area and weakened the adhesion performance. Gel system 2 had a dense grid texture, and each crosslinking formed a whole, resulting in higher cohesion strength. The surface had no obvious protrusions. The overall flatness and roughness were low (Within the ellipse in Figure 5b). The adhesion area increased, and the formed adhesion force became high. The whole Gel system 3 had crosslinking. However, the gel network structure of this system was sparse. The mesh spacing was large. Part of the connection was weak and had the tendency to fracture (Within the ellipse in Figure 5c). The mesh density was obviously lower than the other two types, while the strength was obviously lower. The larger hole-like structure significantly reduced the adhesion area, resulting in poor adhesion performance.

Based on the above experimental analysis, the skeleton density and the degree of networking of the gel system constituted the main factors affecting the strength of the gel system. The micro-surface roughness of materials affects their adhesion capability by influencing the adhesion area. Because of its excellent microstructure, Gel system 2 had the characteristics of high strength, high adhesion, and high plugging compared to Gel systems 1 and 3.

### 2.3. Optimization of the Plugging Capacity of Gel Plugging Agent by Evaluation Index Method

Although the visual code method and the breakthrough vacuum method are simple and easy to use, they only pay attention to the strength of the gel body and neglect the adhesion energy and erosion resistance of the gel to the reservoir pore throat, which eventually leads to the deviation from the results obtained for actual applications. Using a sand-filling pipe or core to simulate the reservoir to conduct profile control plugging displacement test of the gel plugging agent, the displacement test is the closest evaluation method to field conditions. However, this method is complicated and not suitable for the mass selection of gel systems. The morphological observation of the gel/sand mixture and SEM analysis can be operated easily and visually, which can not only effectively compare the strength of the gel adhering to the rock wall, but can also reflect the compatibility of gel and sand. This method can only be used for qualitative comparison and lacks quantitative criterion, due to which it cannot be used as the optimal selection standard. Therefore, the current evaluation method for screening gel systems is not perfect. It is necessary to establish an evaluation method for the gel plugging agent that is simple in operation, quick in experimentation, and accurate in its evaluation of the gel plugging agent.

Considering the strength and adhesion strength of the gel for plugging, the evaluation index method of gel plugging capacity is established to make up for the deficiency of the current indices for evaluating the plugging capacities of gels. The evaluation indices for the plugging capability of the three gel systems were tested and calculated. The strength of gel plugging agents was tested with stainless steel porous mesh with 80 mesh and a thickness of 2.0 mm. The adhesive strength test of the gel plugging agent adopted the reservoir rock slice with a permeability of about 5 μm^2^. The corresponding experimental results are presented in Table 3.

Based upon the comparison of the results presented in Table 3, it can be seen that the cohesion strength and adhesion strength of Gel system 1 were 90 KPa and 190 mN, respectively, both of which were the best. However, the gel system was easy to be damaged by shear, with a degree of damage (*λ*) of 387 N/m^2^, which was the weakest among the studied gel systems. Gel system 2 and Gel system 3 had similar bulk strength and degree of damage; however, Gel system 2 had better adhesion capability. Compared with Gel system 1, Gel system 2 had weak cohesion strength and adhesion capacity. However, the introduction of a resorcinol crosslinking agent forms a higher-strength interpenetrating network structure [32], which improves the resistance to shear damage of the gel system. According to the evaluation index of the plugging capability of the gel plugging agent in different gel systems, the gel system suitable for reservoir profile control and plugging is Gel system 2.

### 2.4. Comparison of Profile Control Capability of the Gel Plugging Agent

**Figure 6:**
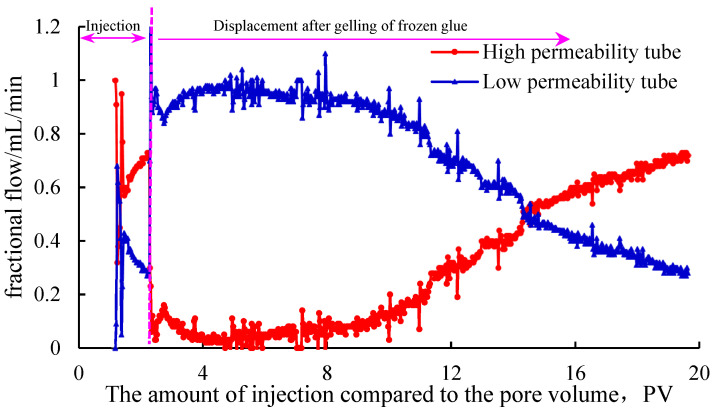
Variation in injection flow rate of Gel system 1 double tube parallel heterogeneous profile control.

**Figure 7:**
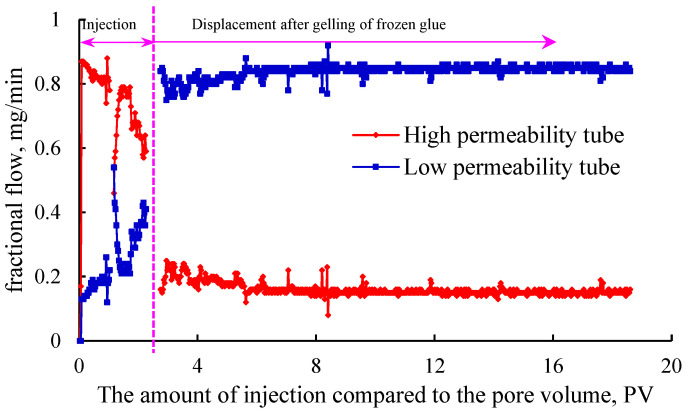
Variation in injection flow rate of 2 double-tube parallel heterogeneous profile control in gel system.

Using a sand-filled pipe or core to simulate the reservoir for the profile controlling of the gel plugging agent, the plugging displacement test is the most suitable testing method. However, this method is complex in operation and not suitable for screening gel systems in large quantities. Therefore, the method can be used as the final verification method for the indoor plugging control of the selected gel systems. According to Equation (1), the permeability of sand-filling pipe filled with 20–80-mesh and 80–120-mesh quartz sand was 8.5 μm^2^ and 1.3 μm^2^, respectively. The high and low permeability reservoirs of heterogeneous reservoirs were simulated in parallel to evaluate the plugging and profile control capability of Gel system 1 (prefield system) and Gel system 2 (optimized field system). The variation curve of the injection flow rate of double-pipe parallel heterogeneous profile control injection for displacement after plugging with the gel plugging agent is shown in Figure 6 and Figure 7.

It can be seen from Figure 6 and Figure 7 that in the gel injection stage, both Gel systems 1 and 2 entered the high permeability tube in large quantities. After the gel system was crosslinked into the gel, the water injection displacement erosion was carried out. In the initial stage, both Gel systems 1 and 2 could reverse the injection flow rate of the high and low permeability pipes, and the liquid ratio of the high and low permeability pipes reached 1:9, respectively, indicating that good profile control was achieved.

It can be seen from Figure 6 that with the water flooding and flushing, when the high and low permeability sand-filling pipes of Gel system 1 were injected and displaced to 8 PV, the difference in the partial flows gradually decreased, and the partial flows of the high and low permeability pipes began to turn over for the second time at 14.3 PV, indicating that the profile control effect was invalid at this time. At 20 PV, the flow ratio of the high and low permeability pipes reached 7:3, and the profile-controlling effect was completely lost. The experimental results also explained the problems that the plugging effect of Gel system 1 was not ideal and the effective period of the plugging was short. Compared with the results presented in Figure 7, it can be seen that the high and low permeability sand-filling pipe of Gel system 2 for plugging control had good profile control and diversion effect, and the comprehensive plugging control effect was superior to that of Gel system 1.

By comparing Figure 6 and Figure 7, it can be seen that Gel system 2 had better profile control and plugging effect than Gel system 1, especially in long-term profile control and plugging capability. The experimental results were consistent with the evaluation index of the plugging capability, showing the feasibility and effectiveness of the method.

### 2.5. Field Tests

**Figure 8:**
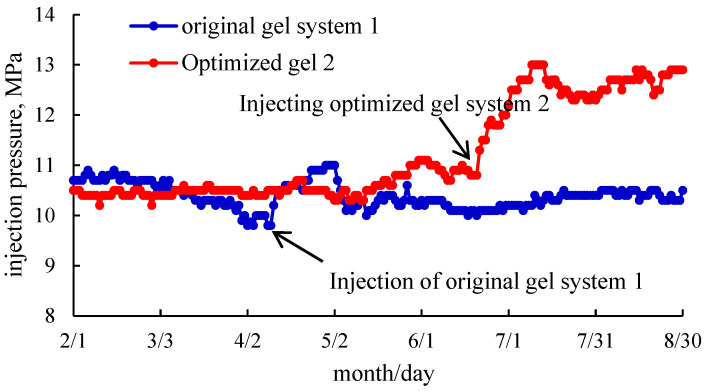
Comparison curve of field test before and after optimization.

Two water injection wells (Ngs (6(1)) and Ngs (6(2))) at the same horizon and similar reservoir properties were selected to carry out the profile control before and after the optimization of the gel system. Figure 8 shows the changes in the pressure curve for the two wells before and after the injection of the profile-controlling agent.

It can be seen from Figure 8 that the pressure of the water injection well injected with the original Gel system 1 increased from 9.8 MPa to 11 MPa since 9 April, indicating that the profile-controlling agent had achieved the profile-controlling effect. However, after 28 days of water injection, the pressure decreased rapidly after the breakthrough, and the final pressure was maintained at the same level as the injection pressure before profile control, indicating that the profile-controlling effect was lost. The pressure of water injection wells injected with the optimized Gel system 2 began to increase from 20 June (up to 13 MPa), and the subsequent pressure decreased slightly though it remained at about 12.5 MPa, indicating that the injection of the profile-controlling agent achieved a good profile-controlling effect and maintained the subsequent stable plugging effect. Gel system 2 was 2.5 times the plugging rate of Gel system 1, while the effective plugging time was more than 60 days. The field test results were consistent with the conclusions obtained by comparing the plugging capabilities.

## 3. Conclusions

1. In the present work, gel system 1 (3000 mg/L HPAM + 0.3% phenolic resin + 0.3% organic chromium crosslinking agent + 0.15% crosslinking accelerator) was screened from three different systems using the conventional breakthrough vacuum method and visual code method. Based on field tests and profile-controlling displacement plugging experiments, the screened gel was easy to separate from the reservoir, resulting in poor plugging capacity. The evaluation effect (strength code: H, degree of breakthrough vacuum: 0.094 MPa, indicating favorable gel performance) was different from the actual plugging capacity (after plugging, the permeability was 2.23 × 10^3^ μm^2^, and the breakthrough pressure gradient was 14.77 MPa/m, indicating poor gel performance). The results showed that the adhesive capability and erosion resistance of the gel to the reservoir pore throat were neglected in the conventional method, and the evaluation angle was found not to be comprehensive enough. The plugging capacity test of the gel plugging agent is cumbersome and not suitable for on-site rapid detection.
2. After analyzing the current laboratory evaluation procedure and summarizing the controlling factors of the plugging capacity of the gel plugging agent, a new evaluation method for the plugging effect of the gel plugging agent was established. Based on this, the gel plugging capability evaluation index method (with the correlation of $\Gamma =\frac{\delta \times \eta }{\lambda }$) was constructed, which comprehensively considered the gel strength, gel adhesion capability, shear resistance, and long-term anti-dehydration performance. The experimental results showed that the evaluation results of the proposed method were consistent with the experimental results of profile control and displacement plugging.
3. A new gel system (3000 mg/L HPAM + 0.3% phenolic resin + 0.15% Resorcinol + 0.3% hexamethylenetetramine + 0.15% crosslinking accelerator) was selected by the newly established method, and its comprehensive evaluation index was found to be 8.97 Pa^2^. Compared with the old system, the failure of profile control gradually occurred when 8 PV was displaced. The new system could achieve a 1:9 ratio of high and low permeability tubes, and the effective period was more than 20 PV. Moreover, the profile control and the plugging capability were obviously improved.
4. A field in the Shengli oilfield was selected for the verification of the experimental results. The gel system optimized by the evaluation method achieved an injection pressure of up to 13 MPa, with a plugging duration exceeding 60 days. The injection pressure of the new system was 2.5 times that before the optimization. The effective plugging period was prolonged to more than two times that of the original system. The field test results were consistent with the plugging capacity evaluation index. To sum up, the newly established method is more scientific and practical, and can provide a reference for screening and evaluating batches of gel plugging agent systems under actual reservoir conditions.

## 4. Materials and Methods

### 4.1. Materials

Resorcinol, hexamethylenetetramine, and ammonium chloride (as the crosslinking accelerator) were analytically pure, and obtained from Sinopharm Chemical Reagent Co., Ltd., Shanghai, China. Phenolic resin ((C_6_H_6_O)_n_.(CH_2_O)_n_; molecular weight of 12,000; alkali number: 20 mg KOH/g), urea–formaldehyde resin ((C_2_H_6_N_2_O_2_); molecular weight of 15,000; alkali number: 50 mg KOH/g), organic chromium crosslinking agent (Cr(C_4_H_6_O_4_)_3_), and partially hydrolyzed polyacrylamide (HPAM; molecular weight of 22 million; degree of hydrolysis of 22–24%) were obtained from the construction site of a block in Shengli Oilfield, Sinopec, Dongying, China. The simulated formation water with a salinity of 20,262 mg/L is prepared in the laboratory, and the specific formula is as follows: Table 4.

### 4.2. The Traditional Test Method of Gel Performance

#### 4.2.1. Visual Code Method [33]

The sample tube was turned upside down to observe the form of a gel in the tube to visually evaluate the strength of the gel. The gel-forming time was determined and the gel strength was adjudged according to the strength code (Table 5) [17]. This method is simple, convenient, and applicable to a wide range of conditions, but the test results are easily affected by the observation of the test container and the tester.

#### 4.2.2. Vacuum Breakthrough Method

**Figure 9:**
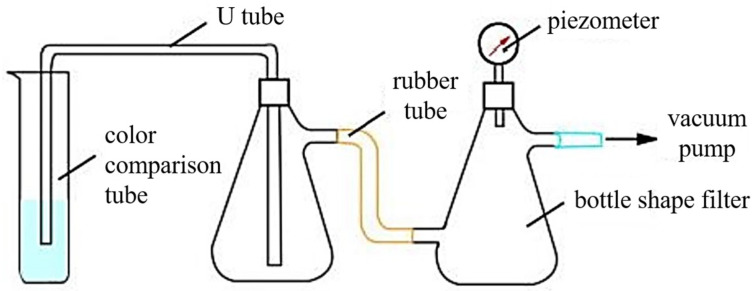
Schematic of the setup used to measure the strength using the breakthrough vacuum method.

The specific experimental device is as shown in Figure 9. The colorimetric tube filled with the formed gel was connected according to the schematic shown in Figure 9. When the vacuum pump was activated and the air broke through the gel, the vacuum on the vacuum gauge increased to the maximum reading (the breakthrough vacuum (BV)). The pressure gauge showed 0 MPa before use. Each sample (or condition) was measured in parallel 3 times, and the average value was used for the analysis (atmospheric pressure at the time of measurement was 0.1 MPa). The larger the BV value, the higher the strength.

#### 4.2.3. Adhesion Sand Test

The adhesion experiment of the gel and sand was carried out to observe the adhesion capability of the system. In the plugging capacity test of the gel plugging agent, the sand-filling pipe was injected with clean water. The advection pump was closed after 16 PV of water was stably displaced. The sand-filling tube was removed, and the mixture of quartz sand and gel was taken out at the 10 mL outlet. The mixture was clamped with tweezers to observe the adhesion of gel and sand.

#### 4.2.4. Cryo-SEM Test of Gel

The microstructure of the gel was observed using scanning electron microscopy (SEM). SU8010 scanning electron microscope used in this experiment was provided by Hitachi High-Tech Analytical Science Co., Ltd., Ibaraki Japan. [34]. First, the gel sample was pre-treated. Liquid nitrogen was used to attain a low temperature of −160 °C. The pressure around the sample was reduced to sublimate the frozen water in the gel so that the water would not crystallize during the solidification process to form glassy water. This is important to avoid the increase in the volume of water due to expansion, which can damage the original structure of the gel in the aqueous solution [35]. The dried polymer mesh was carefully placed on the conductive tape for conductive spraying, and finally, the micro-topography analysis was performed using a scanning electron microscope. The Adhesion Sand Test and Cryo-SEM test of the gel can only qualitatively analyze the adhesion ability, and cannot be quantitatively tested.

### 4.3. Experimental Method of Profile Control Plugging Displacement of Gel Plugging Agent

**Figure 10:**
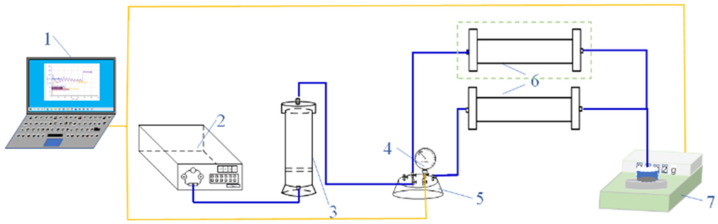
Schematic of the gel plugging agent’s profile-controlling plugging displacement experimental device. 1—data acquisition system; 2—constant-flux pump; 3—intermediate container; 4—pressure sensor; 5—six-way valve; 6—sand-filling pipe; 7—electronic balance.

Using the custom-made displacement device, the plugging capability of the gel plugging agent was analyzed to simulate the plugging effect of the gel system under reservoir conditions [36,37,38,39]. The profile-controlling capability of the gel plugging agent was tested to verify the feasibility of the comprehensive evaluation index of the plugging effect. The flow diagram of the unit is shown in Figure 10. A 20 cm long and 2.5 cm diameter sand-filling pipe was filled with quartz sand to simulate the sandstone reservoir. Sand-filled tubes filled with 20–80-mesh quartz sand simulated the high-permeability reservoirs (4000~5000 mD), whereas sand-filled tubes filled with 80–120-mesh quartz sand simulated the low-permeability reservoirs (400~500 mD).

#### 4.3.1. Plugging Capacity Test of the Gel Plugging Agent

First, a sand-filling pipe was filled with 20–80-mesh quartz sand. The dry weight was determined, and the experimental device was connected according to Figure 10. Clean water was injected at 1 mL/min until the pressure at the injection end stabilized [40]. The pressure was recorded, and the sand-filling pipe was removed to measure the wet weight. The permeability [41] (Kw) and pore volume (PV) were calculated. The sand-filling tube was reconnected, and after injecting 1 PV gel system at the speed of 1 mL/min, the sand-filling tube was removed. Both the ends of the tube were sealed and placed in the thermostat that was set to the gel-forming temperature. After the gel system was gelled, the sand-filling pipe was taken out, and placed into the displacement device, where clean water was injected at the flow rate of 1 mL/min. The amount of injected clean water and the injection pressure were recorded. Finally, the plugging rate and the breakthrough pressure gradient were calculated.

Permeability:

(1) $$K_{w}=\frac{Q\mu_{w}\Delta L}{10A\Delta P}$$

where $K_{w}$ is the water phase permeability (μm^2^), Q is the liquid phase flow (cm^3^/s), $\mu_{w}$ is the fluid viscosity (mPa·s), ΔL is the core length (cm), A is the core’s cross-sectional area (cm^2^), and ΔP is the pressure difference between the injection and outlet ends (MPa).

Plugging rate [23]:

(2) $$\gamma =\frac{k_{1}-k_{2}}{k_{1}}\times 100$$

where γ is the core plugging rate (%), $k_{1}$ is the initial water phase permeability (${\mu m}^{2}$), and $k_{2}$ is the permeability of the water phase after plugging (${\mu m}^{2}$).

Breakthrough Pressure Gradient [19]:

(3) $$p_{m}=\frac{p_{\max}}{L}$$

where $p_{m}$ is the breakthrough pressure gradient (MPa/cm), $p_{\max}$ is the maximum breakthrough pressure (MPa), and L is the length of the sand-filling tube (cm).

#### 4.3.2. Profile-Controlling Capacity Test of Gel Plugging Agent

The sand-filling pipe was replaced with two parallel sand-filling pipes filled with quartz sand of 20–80 meshes and 80–120 meshes, respectively. The other experimental parameters and methods were the same as the plugging capacity test of the gel plugging agent.

### 4.4. Construction of the Evaluation Method for Plugging Effect of Gel Plugging Agent

#### 4.4.1. Body Strength Test Method

**Figure 11:**
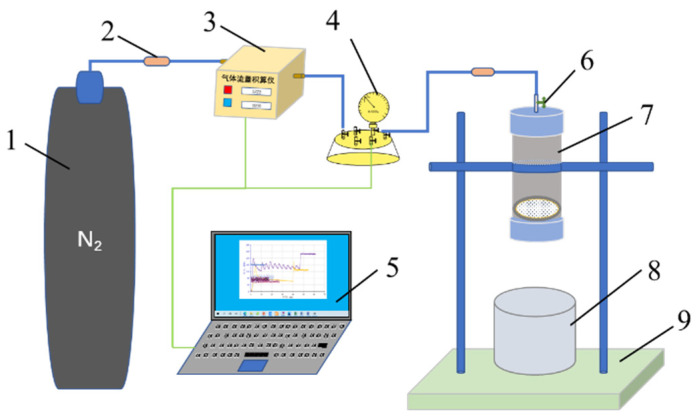
Schematic of the experimental device for cohesion strength test of the gel plugging agent. 1—nitrogen cylinder; 2—check valve; 3—gas flow totalizer; 4—pressure sensor; 5—gas flow control and pressure acquisition system; 6—valve; 7—gel strength test container; 8—sample collection container; 9—bracket table.

Besides good cohesion strength, the shear resistance of gel plugging agents in reservoir throats is also one of the important parameters that help realize a good plugging effect. By simulating the process of the gel plugging agent breaking through the reservoir pore throat, an experimental device for testing the cohesion strength of the gel plugging agent is designed. The schematic of the device is shown in Figure 11. The cohesion strength of the gel plugging agent is characterized using the pressure difference when the gel plugging agent breaks through the simulated pore throat.

The experimental device for testing the strength of the gel plugging agent mainly consisted of a pressure supply system (represented by 1, 2, 3, and 4 in Figure 11), a data acquisition system (represented by 5 in Figure 11), and a simulation system for the gel plugging agent to break through the pore throat (represented by 6 and 7 in Figure 11). In order to avoid the influence of pressure difference on the experimental results, the gas flow integrating instrument in the pressure supply system (represented by 3 in Figure 11) was designed to control the rate of gas output, and the rate of increase in pressure in the gel strength test vessel (represented by 7 in Figure 11).

**Figure 12:**
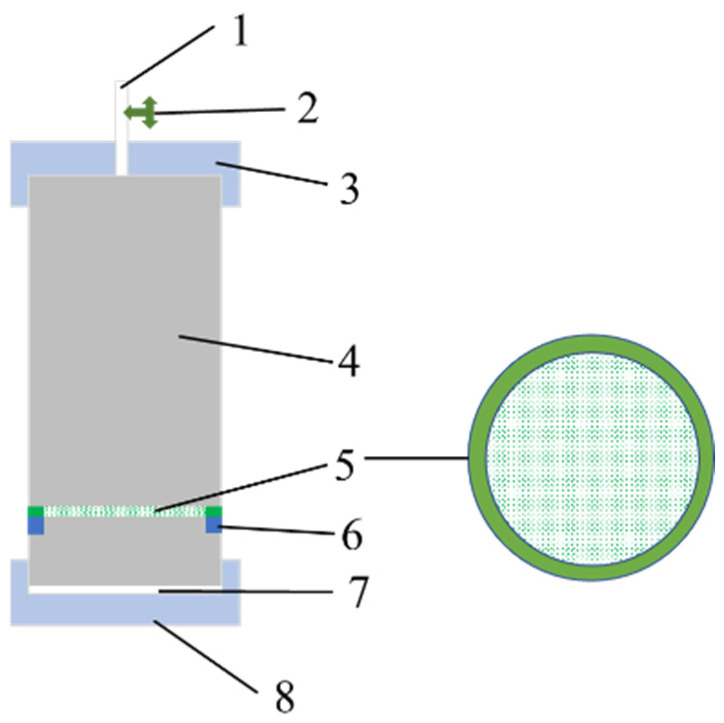
Schematic of the gel strength test container. 1—opening pipeline joint; 2—valve; 3—container top cover; 4—container cavity; 5—porous mesh; 6—partition plate in the vessel; 7—liner; 8—container lower lid.

The gel strength test container is key to the device used for evaluating the strength of the gel plugging agent. The schematic of the container is shown in Figure 12. The vessel cavity (represented by 4 in Figure 12) was made up of transparent plexiglass, thus meeting the pressure requirements, which can visually observe the gel plugging agent breakthrough process. The porous mesh (represented by 5 in Figure 12) was a regularly arranged mesh. Different mesh sizes can be used to simulate different reservoir permeabilities. The materials can be stainless steel or organic glass. The porous mesh can also be replaced by the cemented core sheet or the core sheet cut and processed by the on-site reservoir.

The inner lining (represented by 7 in Figure 12) corresponding to the lower cover of the container was made up of polytetrafluoroethylene. During the experiment, a layer of Vaseline was applied to reduce the adhesion between the gel and the lower cover and reduce the experimental error.

Since the transfer of a gel plugging agent to any strength-measuring device will cause some damage to its performance, the measured value is usually not representative of the real gel strength [19,42]. Therefore, the gel plugging agent is designed to first gel and crosslink in the container used to measure the gel strength, and then, the strength test is performed. The experimental device used for determining gel strength is shown in Figure 11. The detailed experimental procedures are outlined as follows:

1. The solution of the gel system was poured into the container used to measure the strength of the gel. The liquid level was 2 cm higher than the porous mesh.
2. The container was sealed and placed in the thermostat box, which was set to the gelling temperature.
3. After the gel system was gelled, the container was taken out. The upper cover valve was opened, emptied, and fixed on the bracket table. The experimental device was connected as shown in Figure 11.
4. The lower cover of the container was opened. The gas flow rate was set to be 20 mL/min. The pressure reading was recorded. The maximum pressure value was collected when the gel broke through, which was the body strength of the gel plugging agent.

#### 4.4.2. Adhesion Strength Test Method

**Figure 13:**
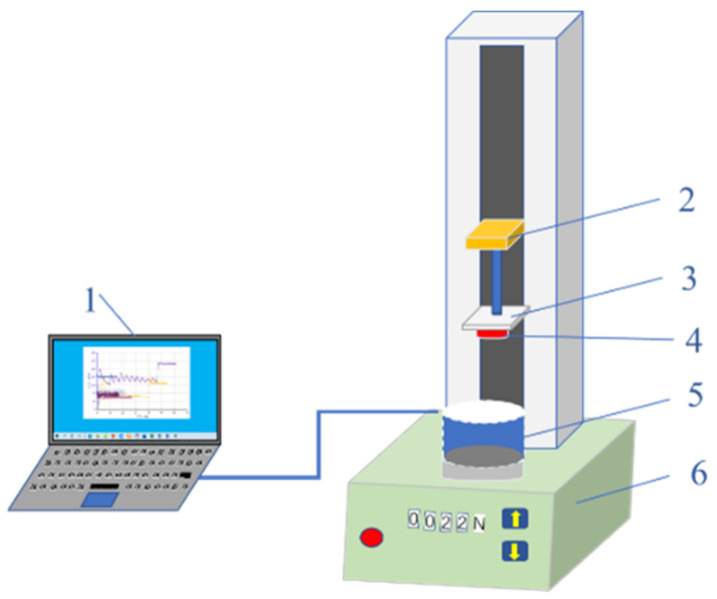
Schematic of the test device used for adhesive strength test of gel plugging agent. 1—data acquisition system; 2—upper and lower lifting platform; 3—plate of lifting table; 4—core slice; 5—transparent sample cup; 6—precision tensile testing machine.

The gel plugging agent has good adhesion to the pore throat of the reservoir, which can improve erosion resistance. Adhesion is another necessary condition for achieving a good plugging effect. The schematic of the test device used for evaluating adhesion between the gel plugging agent and the reservoir pore throat wall is shown in Figure 13. The core slice (represented by 4 in Figure 13) adopted the standard cylindrical slice (a diameter of 2.54 cm) of stratum core, with a thickness of 5–10 mm. If the formation core was not available, it could be replaced by other rock slices with similar material and permeability.

The adhesive strength of the gel plugging agent was characterized using the maximum tensile force between the pore throat wall and the gel plugging agent per unit area. The adhesive strength is given by Equation (4).

(4) $$\eta =\frac{F}{S}$$

where *η* is the adhesive strength of the gel plugging agent to reservoir pore throat (N/m^2^), *F* is the maximum tensile force when the gel plugging agent is peeled off from the core sheet (N), and *S* is the surface area under the core (m^2^).

The adhesive strength of the gel plugging agent was measured using the experimental device shown in Figure 13. The detailed experimental procedures are outlined as follows:

1. The gel system solution was poured into a transparent sample cup to a height of 4–6 cm. The cup was sealed and placed in a constant-temperature box that was set to the gelling temperature.
2. After the gel system was gelled, the sample cup was taken out. The cover of the sample cup was removed and placed on the sample table of the precision tensile testing machine.
3. The cutting flat core piece was bonded to the lifting platform plate, and the experimental device was connected as shown in Figure 13.
4. The lifting platform was controlled to drop lower until the lower end of the core slice was in close contact with the gel and maintained for a period of 5–10 min.
5. The lifting speed of the lifting platform was set to 2 mm/min. The value of the tension was collected when it started to rise. The maximum tension value (F) was collected, and the adhesion strength of the gel plugging agent was calculated by Equation (4).

#### 4.4.3. Method for Measuring the Degree of Damage

Dehydration of a gel plugging agent significantly reduces its plugging capacity, which is an important reason for shortening the effective period of profile control and water plugging in oilfields [43]. The process of migration and shearing will increase the dehydration of the gel. The rate of dehydration of the gel was adopted to represent the degree of damage to the gel. The detailed experimental procedures are outlined as follows:

1. After the strength test of the gel body, the gel sheared by the porous mesh was taken. The mass of the gel was *m*_1_ (g). The gel was placed in a suitable cover screw glass test tube and sealed.
2. The sealed gel was placed in the constant-temperature box for 48 h, which was set to the reservoir temperature.
3. The screw glass test tube was removed. The amount of water from the gel was noted down, and the mass was observed as *m*_2_ (g). The gel damage index is calculated using the expression $\lambda =\frac{m_{2}}{m_{1}}\times 100$.

### 4.5. Construction of the Comprehensive Evaluation Index of Plugging Effect

The plugging capability of the gel plugging agent is positively correlated with the cohesion strength and adhesion strength of the gel plugging agent. The plugging capability is negatively correlated with the degree of loss. Therefore, the comprehensive evaluation index (Γ) is defined to evaluate the plugging effect of the gel plugging agent on the reservoir. The comprehensive evaluation index is given by Equation (5).

(5) $$\Gamma =\frac{\delta \times \eta }{\lambda }$$

## Figures and Tables

**Table 2:** Test results for the plugging performance of the gel systems.

Gel System	Permeability Before Plugging × 10^3^, μm^2^	Permeability After Plugging × 10^3^, μm^2^	Permeability After Displacement of 16 PV × 10^3^, μm^2^	Plugging Rate, %	Plugging Rate After Displacement of 16 PV, %	Breakthrough Pressure Gradient, MPa/m
Gel system 1	8493.6	2.23	1.98	99.97	99.98	14.77
Gel system 2	8495.8	0.90	1.45	99.99	99.98	36.57
Gel system 3	8542.1	6.41	46.3	99.92	99.45	4.70

**Table 3:** Evaluation index of plugging capability of gel plugging agent in different gel systems.

Gel System	Body Strength *δ*, KPa	Adhesion Strength *η*, N/m^2^			Degree of Injury *λ*, Dimensionless	Plugging Capacity Evaluation Index *Γ*, Pa^2^
		Adhesive Force, mN	Core Slice Diameter, cm	*η*, N/m^2^		
Gel system 1	90	190	2.5	387.3	6.4	5.45
Gel system 2	66	140	2.5	285.4	2.1	8.97
Gel system 3	63	60	2.5	122.3	2.3	3.35

**Table 4:** Formula table of simulated formation water.

Ionic Types	Ca^2+^	Na^+^	Mg^2+^	Cl^−^	SO_4_^2−^	HCO_3_^−^
ion concentration, mg/L	1022	6422	259	12,326	31	202

**Table 5:** Code standards for gel strength.

Grade	Strength Classification Standard
A	No continuous gel formation was detected: the viscosity of the gelling system was the same as that of the same concentration of polymer solution without a crosslinking agent, but sometimes there might be some unconnected gel blocks with great viscosity in the system.
B	Highly flowable gel: The viscosity of the gel system is slightly higher than that of a polymer solution of the same concentration without a crosslinking agent.
C	Flowing gel: When the sample bottle is inverted, most of the gel flows to the cap.
D	Medium flow gel: Only a small portion (10~15%) when the vial is inverted vertically. The gel does not easily flow to the cap (typically described as long tongue type gel).
E	Difficult to flow gel: When the sample bottle is vertically inverted, the gel flows very slowly to the cap or a large part (>15%) does not flow to the cap.
F	Highly Deformed No Flow Gel: Gel does not flow to the cap when the vial is inverted vertically.
G	Moderate deformation non-flowing gel: When the sample bottle is vertically inverted, the gel is deformed downward to about half of the position.
H	Slight deformation does not flow gel: When the sample bottle is vertically inverted, only the surface of the gel is slightly deformed.
I	Rigid gel: When the sample bottle is inverted vertically, the surface of the gel does not deform.

## Data Availability

The raw data supporting the conclusions of this article will be made available by the authors on request.

## Abbreviations

The following abbreviations are used in this manuscript:

GSR	gel strength rank index method
BV	breakthrough vacuum
HPAM	partially hydrolyzed polyacrylamide
PV	pore volume
